# Scrub Typhus Outbreak in Chonburi Province, Central Thailand, 2013

**DOI:** 10.3201/eid2402.171172

**Published:** 2018-02

**Authors:** Wuttikon Rodkvamtook, Narupon Kuttasingkee, Piyada Linsuwanon, Yutthapong Sudsawat, Allen L. Richards, Maneerat Somsri, Noppadon Sangjun, Chien-Chung Chao, Silas Davidson, Elizabeth Wanja, Jariyanart Gaywee

**Affiliations:** Royal Thai Army Component—Armed Forces Research Institute of Medical Science, Bangkok, Thailand (W. Rodkvamtook, N. Kuttasingkee, Y. Sudsawat, M. Somsri, N. Sangjun, J. Gaywee);; US Army Medical Directorate—Armed Forces Research Institute of Medical Science, Bangkok (P. Linsuwanon, S. Davidson, E. Wanja);; Naval Medical Research Center, Silver Spring, Maryland, USA (A.L. Richards, C.-C. Chao)

**Keywords:** Scrub typhus, *Orientia tsutsugamushi*, seroprevalence, vector-borne infections, Thailand, bacteria

## Abstract

Investigation of a scrub typhus outbreak in Thailand during September 2013 found that 9.1% of Thai soldiers and 11.1% of residents living in areas surrounding training sites had antibodies against the causative agent, *Orientia tsutsugamushi*. Sequence analysis of *O. tsutsugamushi* from rodents and chiggers identified 7 genogroups and 3 genotypes.

Although scrub typhus is one of the leading causes of acute febrile illnesses (AFI) in the Asia-Pacific region, with an estimated 1 million cases annually (*1*,*2*), its overall molecular epidemiology remains unclear. Scrub typhus occurs when humans are bitten by parasitic larvae of trombiculid mites (chiggers) harboring the bacterium *Orientia tsutsugamushi*. Chiggers primarily infest mammals, such as rodents, that dwell in tall grasses and scrub vegetation; thus, activities such as camping and hiking can lead to scrub typhus (*3*,*4*). Although scrub typhus is treatable with doxycycline if diagnosed early, the median fatality rate among scrub typhus patients is 6%, and the fatality rate may reach 20% among untreated patients (*5*,*6*). No effective vaccine is currently available because of high variation in *O. tsutsugamushi* immunogenicity (*7*,*8*).

Scrub typhus outbreaks are problematic for the Royal Thai Army (RTA), particularly troops conducting field training in central and northeastern regions of Thailand. In a 2002 scrub typhus outbreak reported among RTA soldiers who trained in fields located in Bo Thong District, Chonburi Province, 9.8% of exposed soldiers showed seropositivity to scrub typhus group (STG) antigens (*9*). In 2013, an unusual pattern of AFI emerged among soldiers who conducted military operations at Si Racha, 89 km from Bo Thong. We describe the scrub typhus outbreak among RTA soldiers in 2013 and demonstrate successful integration of a control program designed with the Armed Forces Research Institute of Medical Science (AFRIMS), the AFRIMS-Nawamintharachini model, to provide more accurate assessment of potential health risks to soldiers conducting training exercises in scrub typhus–endemic areas.

## The Study

**Figure 1:**
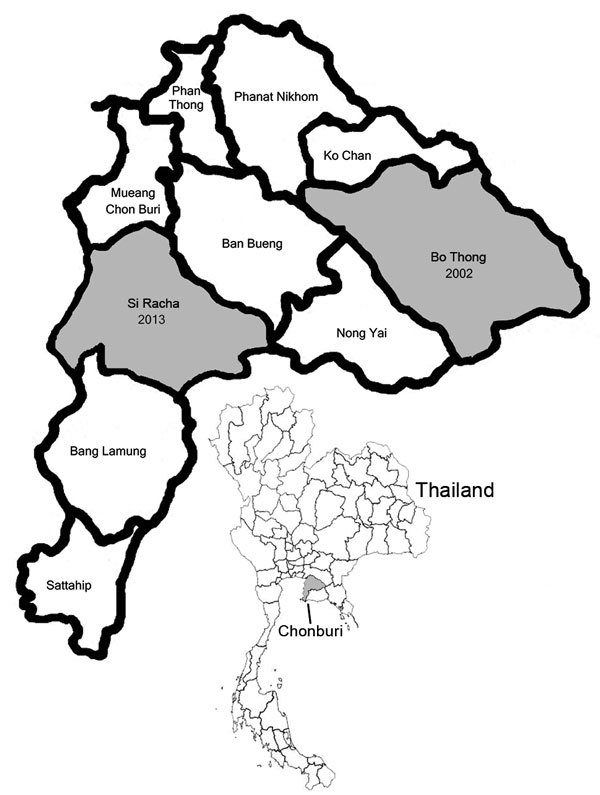
Chonburi Province, Thailand, showing scrub typhus outbreak areas in in Bo Thong district in 2002 and Si Racha district in 2013 (gray shading). Inset shows location of Chonburi Province in Thailand.

During September 2013, a group of 110 RTA soldiers conducted operational field training near the Bang Phra Navy Agriculture Center in Si Racha (Figure 1). Two to 3 weeks after training, 10 soldiers experienced sudden onset of high-grade fever with clinical symptoms including intense headache, chill, and retroorbital eye pain. Eight of these soldiers were hospitalized at Fort Nawamintharachini Hospital, and the other 2 were admitted to other hospitals in Chonburi. Physical examinations revealed that 37.5% had necrotic lesions similar to eschar. Serum samples were send to AFRIMS for diagnostic tests for the most likely pathogens: PCR for dengue viruses and indirect immunofluorescent assay and dot ELISA for rickettsioses (*10*,*11*) ( Table 1).

**Table 1:** Clinical manifestations and laboratory test results for scrub typhus in 10 RTA soldiers who trained in Si Racha District, Chonburi Province, Thailand, 2013*

ID	Age, y	Clinical signs and symptoms					Symptom onset to collection, d†	Test results for *O. tsutsugamushi*, IgM/IgG‡						
Fever	Eschar	Headache	Chill	Eye pain	IFA				Dot-ELISA		
					1	2	3		1	2	3
P01	25	+	+	+	+	+	3	Neg/Neg	1,600/1,600	1,600/1,600		−/+	+/+	+/+
P02	22	+	−	+	+	−	5	200/50	1,600/50	1,600/50		+/+	+/+	+/+
P03	25	+	+	+	+	−	5	200/200	1,600/3,200	1,600/3,200		+/+	+/+	+/+
P04	23	+	−	+	+	−	5	200/100	3,200/800	3,200/800		+/+	+/+	+/+
P05	21	+	−	+	+	−	5	200/100	3,200/800	3,200/800		+/+	+/+	+/+
P06	21	+	−	+	+	+	5	800/100	6,400/3,200	6,400/3,200		+/+	+/+	+/+
P07	21	+	−	+	−	−	8	ND	1,600/100	6,400/6,400		ND	+/+	+/+
P08	22	+	−	−	+	−	9	ND	800/50	800/50		ND	+/+	+/+
P09	45	+	−	+	−	−	23	ND	ND	1,600/1,600		ND	ND	+/+
P10	21	+	+	+	+	+	12	ND	ND	3,200/3,200		ND	ND	+/+

*ID, patient identification number; IFA, indirect fluorescence antibody assay; ND, not determined; + positive; –, negative.  †Time from onset of signs and symptoms to first blood collection. ‡Karp, Kato, and Gilliam genotypes. Tests were performed on September 27 (round 1), October 4 (round 2), and October 7 (round 3). Positivity cutoff titer for initial screening was 1:50 and the diagnostic criteria were established as a >4-fold increase in the titer of IgM or IgG in paired serum samples. If only 1 serum sample was available, titer of 1:50 to <1:400 was determined as recent or previous infection and titer >1:400 as active infection.

Serologic analysis showed that all patients had positive antibody titers against STG, suggesting active infection. Patients were treated with oral doxycycline, and fever subsided within 24 h without any sequelae. For the 2 soldiers admitted at different hospitals, the serum samples from the acute and convalescent phases were not available for the assay but were collected for follow-up analysis. Serologic analysis suggested that these soldiers had higher titers of STG IgM and IgG, indicating history of *O. tsutsugamushi* infection. 

To determine the exposure of the troops to disease vectors in the training areas, we identified the serostatus of 100 additional RTA soldiers who conducted field training exercises in the same areas and of 27 residents and workers in the agriculture center. All 127 participants were healthy and afebrile at the time of blood collection. Serum tests of the residents and workers revealed that 3/27 had STG IgG, suggesting high exposure to *O. tsutsugamushi*. According to interviews, 24 soldiers reported experiencing AFI during training. Test results demonstrated that none of these soldiers had *O. tsutsugamushi* infection during the training period. We concluded that seroprevalence of STG antigens among the troops was 9.1% and among residents, 11.1%.

To control the scrub typhus outbreak, AFRIMS applied an established prevention and control program, the AFRIMS-Nawamintharachini model, which was established in 2003 after a scrub typhus outbreak in Bo Thong District. The model describes controlling scrub typhus outbreaks through a surveillance program to clarify vector distribution and rodent population density. The program included developing diagnostic methods, increasing scrub typhus infection awareness, and providing topical insect repellents to soldiers who conduct activities in locations likely to harbor scrub typhus, as well as observing soldiers for 15 days after completing deployment for indications of AFI. We introduced this program in Si Racha after the 2013 outbreak, and since then, no scrub typhus infection has been reported in postdeployment soldiers who trained in previously defined scrub typhus risk areas.

**Figure 2:**
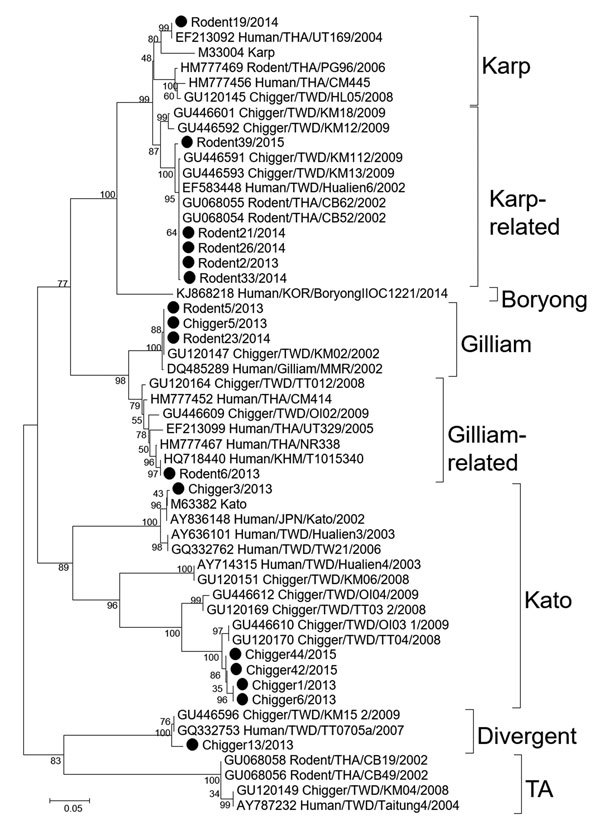
Phylogenetic tree of nucleotide sequences of partial *Orientia tsutsugamushi* 56-kDa type-specific antigen encoding genes obtained from rodents and chiggers in Chonburi Province, Thailand, 2013 (black circles). Tree was constructed by neighbor-joining on the basis of the Kimura 2-parameter model and maximum-likelihood methods using the general time reversible model. Bootstrapping for 1,000 replications was included in all phylogenetic tree constructions. No difference in tree topology was observed among phylogenetic trees constructed by the 2 methods. Nucleotide sequences of chiggers and rodent tissue samples from Thailand have been deposited in GenBank (accession nos. MF431253–MF431268); GenBank accession numbers are provided for reference sequences. Genotypes are indicated at right. Scale bar indicates nucleotide substitutions per site.

Because rodents influence mite population density (*12*,*13*), we surveyed for chiggers and small rodents in these areas to determine the etiology associated with scrub typhus outbreak and evaluate the effectiveness of the prevention program. We conducted surveys in 3 consecutive years (August 2014, November 2015, and August 2016) by following a previously described protocol (*10*). We surveyed areas covering a 10-km radius near training sites with small villages and various vegetation characteristics. We collected rodent liver, spleen, and serum specimens and stored them in liquid nitrogen. We removed chiggers from rodents’ ears and preserved them in 70% ethanol. We slide-mounted approximately one fifth of chigger samples from each rodent for species identification and subjected the remaining chiggers to nucleic acid extraction. We extracted nucleic acid from chiggers and rodent liver and spleen samples using GeneJET Viral DNA/RNA Purification Kit (Invitrogen, Waltham, Massachusetts, USA) and assayed samples for *O. tsutsugamushi* DNA using 47-kDa quantitative PCR and 56-kDa nested PCR (*14*). We bidirectionally sequenced partial sequences of 56-kDa genes (First BASE Laboratories, Singapore) and used BioEdit software (http://bioedit.software.informer.com/7.1/) with the ClustalW algorithm to align nucleotide sequences with the sequences of *O. tsutsugamushi* prototypes and variants retrieved from GenBank. We constructed phylogenetic trees using MEGA software (http://www.megasoftware.net/) (Figure 2).

We captured a total of 45 rodents, accounting for a 7% capture rate. *Bandicota* (n = 5) and *Rattus* (n = 30) spp. rats were the most commonly trapped small mammals (77.8%), followed by ground squirrels (*Menetes berdmorei*, 17.8%) and tree shrews (*Tupaia glis*, 4.4%) (Table 2). Of the small mammals collected, 71.1% (32/45) tested STG seropositive. Most ectoparasites collected from rodents were chigger mites (88.9%), with *Leptotrombidium deliense* (72.1%) the predominant species of the 400 individual chiggers identified. A total of 20% of rodent liver and spleen samples and 15.6% of individual chiggers were PCR positive for *O. tsutsugamushi*. Phylogenetic analyses of the 56-kDa genes of 9 rodent and 7 chigger samples suggested that sequences were clustered in 3 genogroups and were closely related to 3 genotypes (Karp-related, Kato-related, and Gilliam-related genotypes) with 92.6%–99.8% nucleotide identity with reference sequences (Figure 2). Variants from the same clusters showed 94%–98% nucleotide identity with each other. Additionally, phylogenetic trees suggested that *O. tsutsugamushi* strains recovered from chiggers differed from those of rodent hosts, indicating high genetic variation of *O. tsutsugamushi* strains circulating in the survey areas.

**Table 2:** Chigger infestation and *Orientia tsutsugamushi* infection in rodent and shrew species, Chonburi Province, Thailand, 2013*

Family	Species	No. captured (% of total)	No. infested with chiggers, (% of species total)	No. (%) seropositive for STG by IFA	No. (%) positive for OT by PCR
*Muridae*	*Bandicota indica* rat	4 (8.9)	4 (100)	4 (100)	0
	*B. savilei* rat	1 (2.2)	1 (100)	0	1 (100)
	*Rattus rattus* rat	27 (60)	25 (92.6)	27 (100)	7 (25.9)
	*R. exulans* rat	3 (6.7)	1 (33.3)	1 (33.3)	1 (33.3)
*Sciuridae*	*Menetes berdmorei* squirrel	8 (17.8)	7 (87.5)	ND	1 (16.7)
*Tupaiidae*	*Tupaia glis* shrew	2 (4.4)	2 (100)	ND	0
All		45	40 (88.9)	32 (71.1)	10 (22.2)

*IFA, indirect fluorescence antibody assay; ND, not determined; OT, *Orientia tsutsugamushi*; STG, scrub typhus group of antigens.

## Conclusions

High seropositive status for *O. tsutsugamushi* among RTA soldiers and the presence of disease-carrying vectors and rodent hosts trapped in training and surrounding areas indicate that soldiers in these areas are at risk of scrub typhus, with the infection rate estimated at 9%–11%. Military personnel and residents who participate in exercises or travel to these areas in Chonburi should be alerted to the risk for scrub typhus. Appropriate protection, such as proper use of treated uniforms and application of topical insect repellent, are recommended to prevent chigger bites. Environment modification, including vegetation cleanup and trash consolidation, is necessary to control rodent and chigger populations in training location and decrease disease transmission.
